# Association between the triglyceride-glucose index and carotid plaque incidence: a longitudinal study

**DOI:** 10.1186/s12933-022-01683-6

**Published:** 2022-11-15

**Authors:** Yichi Zhang, Zhuchao Wu, Xiaona Li, Jingkai Wei, Qun Zhang, Jianming Wang

**Affiliations:** 1 Department of Epidemiology, Center for Global Health, School of Public Health, Nanjing Medical University, Nanjing, 211166 China; 2 Department of Health Management Center, The First Affiliated Hospital of Nanjing Medical University, Nanjing, 210029 China; 3 Department of Health Management, School of Public Health, Nanjing Medical University, Nanjing, 211166 China; 4 Department of Epidemiology and Biostatistics, Arnold School of Public Health, University of South Carolina, Columbia, SC 29208 USA

**Keywords:** Atherosclerotic cardiovascular disease, Triglyceride-glucose index, Carotid plaque, Cohort study

## Abstract

**Background:**

Carotid plaque and triglyceride-glucose (TyG) index are associated with insulin resistance. However, a highly debated question is whether there is an association between the TyG index and carotid plaque incidence. Thus we performed an in-depth longitudinal study to investigate the relationship between carotid plaque occurrence and the TyG index among Chinese individuals.

**Methods:**

Two thousand and three hundred seventy subjects (1381 males and 989 females) were enrolled and followed up for three years. The subjects were stratified into four groups based on the quartile of the TyG index at baseline. Univariate and multivariate Cox proportional hazard models were conducted to examine the role of TyG played in the carotid plaque. The strength of association was expressed as hazard ratio (HR) and 95% confidence interval (CI).

**Results:**

After three years of follow-up, 444 subjects were detected with newly formed carotid plaque. The overall 3-year cumulative carotid plaque incidence was 18.7%, and the risk of carotid plaque increased with elevated TyG index (*p* < 0.001). The Cox regression analysis showed that males (HR: 1.33, 95% CI: 1.10–1.61), and people with higher systolic blood pressure (HR:1.01, 95% CI: 1.01–1.02), lower high-density lipoprotein cholesterol (HR: 0.68, 95% CI: 0.50–0.93), diabetes (HR: 2.21, 95% CI: 1.64–2.97), and hypertension (HR:1.49, 95% CI: 1.23–1.81) had a significantly increased risk for the carotid plaque formation. Similar results remained in the sensitivity analysis.

**Conclusions:**

The TyG index can be used as a dose-responsive indicator of carotid plaque in the Chinese population. Elderly males with dyslipidemia, diabetes, or hypertension should be more vigilant about their TyG index since they are susceptible to developing carotid plaque. Physicians are encouraged to monitor the TyG index to help identify and treat patients with carotid plaque at an early stage.

**Supplementary Information:**

The online version contains supplementary material available at 10.1186/s12933-022-01683-6.

## Introduction

Despite significant breakthroughs in preventing and treating cardiovascular disorders, atherosclerotic cardiovascular disease (ASCVD), such as coronary artery disease (CAD) and stroke, continues to be the leading cause of death and disability globally [1]. ASCVD was also a leading cause of death and a severe public health problem in China. The incidence and death rates of ASCVD in China were predicted to climb steadily over the following decade due to increasing urbanization and an aging population [2]. Plaque disruption and thrombus formation were the primary causes of cerebral stroke and myocardial infarction, and atherosclerosis constituted an independent risk factor for ASCVD [3]. As a result, the presence of carotid plaque was associated with an elevated incidence of stroke and coronary heart disease [4]. Compared to carotid intima-media thickness (cIMT) of the arterial wall, which reflects the severity of coronary artery atherosclerosis, the presence of carotid plaque predicts a higher risk of carotid atherosclerosis [5, 6]. Early detection of carotid plaque may assist in identifying individuals at increased risk of carotid atherosclerosis and some other acute cardiovascular illnesses who may benefit from carotid revascularization [7], reducing the burden of ASCVDs on the general public.

The carotid plaque burden was rising significantly across the globe. A systematic review showed that carotid plaque was present in 20.2% of Chinese adults between the ages of 30 and 80 [8]. By 2020, approximately 21.1% of the world's population will develop carotid plaque [4]. Carotid plaque was found to be present in about 30% of ischemic stroke cases [9]. The significant disease burden of carotid plaque requires effective preventive strategies. Identifying risk factors associated with carotid plaque can help detect patients with carotid plaque and reduce the disease burden.

Several risk factors have been linked to carotid plaque and the development of ASCVD, most notably insulin resistance (IR), which refers to a phenomenon in which the biological response of insulin is lower than usual. The IR was a crucial intermediate process in metabolic disorders, type 2 diabetes mellitus (T2DM), and ASCVD [10]. The IR was a complex phenomenon that required sophisticated assessment methods, which were unsuitable for large populations. The homeostasis model assessment of IR (HOMA-IR) was widely employed in clinical settings [11]. However, the calculation of HOMA-IR required the determination of fasting insulin levels, which was difficult to achieve in most primary healthcare institutions. Therefore, researchers have proposed potential surrogate markers for HOMA-IR [12].

Recently, a growing body of evidence has revealed a strong correlation between the triglyceride-glucose (TyG) index and the HOMA-IR assessment [13]. Therefore it can be proposed as a simple surrogate of IR. The TyG index was derived using triglyceride (TG) and fasting blood glucose (FBG) values [14]. By combining TG and FBG, the TyG index became a non-insulin-based indicator that is less costly than HOMA-IR to assess IR. Furthermore, a previous study found that the TyG index had greater sensitivity (96.5%) and specificity (85.0%) to detect IR than the gold standard, the hyperinsulinemic-glycemic clamp test [12].

Given the significant carotid plaque prevalence and the fact that both the TyG index and carotid plaque are associated with IR, it was desirable to look into the relationship between the TyG index and carotid plaque. However, the association between the TyG index and carotid plaque incidence remains somewhat controversial, and there was no general agreement about whether the TyG index was substantially related to the incidence of carotid plaque. A study on patients with prediabetes and newly diagnosed type 2 diabetes mellitus (T2DM) showed a positive association between the TyG index and carotid plaque occurrence [15]. Another research has established that an elevated TyG index could raise the likelihood of carotid plaque formation in the overall public [9]. However, Zhao et al. reported no link between these two variables [16].

We found heterogeneity in the distribution of carotid plaque in different subgroups in the literature. A demographic healthcare investigation showed a significant change in the sex difference in the prevalence of atherosclerosis [17]. The most decisive risk factors were age, gender, tobacco smoking, and T2DM history [18]. Given the different distributions of carotid plaque among various subgroups, it is desirable to examine the associations within each distinct subset. Therefore, we conduct a cohort study in a Chinese population to explore the relationship between the TyG index and carotid plaque incidence.

## Methods

### Study population

**Fig. 1:**
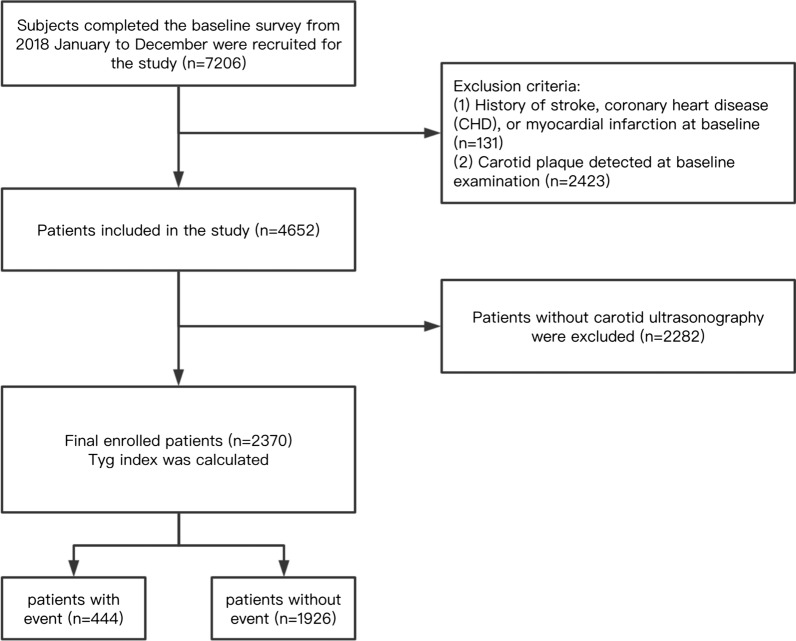
Flow chart of the study population enrollment

We recruited subjects who participated in the annual physical examination at the Health Management Center of Jiangsu Province Hospital from 2018 to 2020. In total, 7206 participants provided written informed consent and finished the baseline survey in 2018. They were qualified and enrolled for the study. After excluding 131 people with a history of stroke, coronary heart disease (CHD), or myocardial infarction at baseline, and 2423 participants detected carotid plaque at the preliminary examination, 4652 individuals remained for follow-up. We ruled out 2282 participants missing carotid ultrasonography data during follow-up, and then 2370 individuals were included in the final analysis (Fig. 1).

### Survey and measurements

All participants were asked to complete a baseline questionnaire survey, physical assessment, laboratory investigations, and a symmetrical carotid ultrasonography inspection. Trained investigators interviewed all participants with a structured questionnaire that enclosed demographic characteristics (age, sex, occupation), and medical history (T2DM, dyslipidemia, hypertension, cerebrovascular disease, and cardiovascular disease). Senior clinicians performed the physical examination, laboratory test, and bilateral carotid ultrasound examination. The relevant data from the first and last physical examinations were compiled and compared for analysis.

### Physical examinations

Body weight (kg) divided by the square of body height (m^2^) was used to determine the body mass index (BMI). Using an automated sphygmomanometer (Omron HEM-7211; Omron Corp., Kyoto, Japan), resting blood pressure was recorded on the brachial artery in the right upper arm while the subject was seated. After a 10-min rest, systolic blood pressure (SBP) and diastolic blood pressure (DBP) were calculated by averaging the last three values with a 2-min gap between the assessments.

### Laboratory examination

After a 12-h fast, 5 mL of blood was drawn by venipuncture in the morning. The blood specimens were centrifuged (3000 r/min, 10 min) after being left at ambient temperature for two hours, and the blood serum was extracted. All measurements were performed by an Olympus AU2700 analyzer (Olympus, Kobe, Japan). Routine biochemical data includes triglyceride (TG), total cholesterol (TC), fasting blood glucose (FBG), low-density lipoprotein cholesterol (LDL-C), and high-density lipoprotein cholesterol (HDL-C). All laboratory tests accomplished the standardization.

### Definitions

Hypertension was defined if a person having systolic blood pressure (SBP) ≥ 140 mmHg or diastolic blood pressure (DBP) ≥ 90 mmHg or a self-declared history of hypertension [19]. Diabetes was defined as FBG levels ≥ 7.0 mmol/L or any self-declared history of diabetes or current drug use for diabetes [20]. Dyslipidemia was defined as those taking oral anti-dyslipidemic drugs, having any self-declared history, or at any one of the following outcomes: TG levels ≥ 1.70 mmol/L, TC levels ≥ 5.18 mmol/L, LDL-C levels ≥ 3.37 mmol/L, or HDL-C level ≤ 1.04 mmol/L [21].

Smoking status was self-reported as current, former, and never smoker. Those who had smoked cigarettes consistently during the past six months were considered to be current smokers. Former smokers were identified as those who stopped smoking for at least 6 months but had smoked at least 100 cigarettes in their entire life. Individuals who never smoked during their lifetime were defined as never-smokers [22].

BMI (kg/m^2^) was used to categorize the weight status into 4 categories: underweight (< 18.5), normal (18.5–24.9), overweight (25.0–29.9), and obese (≥ 30.0) [23]. The TyG index was denoted using the well-developed formula: TyG = Ln [TG (mg/ml) * FBG (mg/ml) /2] [12, 24, 25]^.^

### Atherosclerosis assessment

**Fig. 2:**
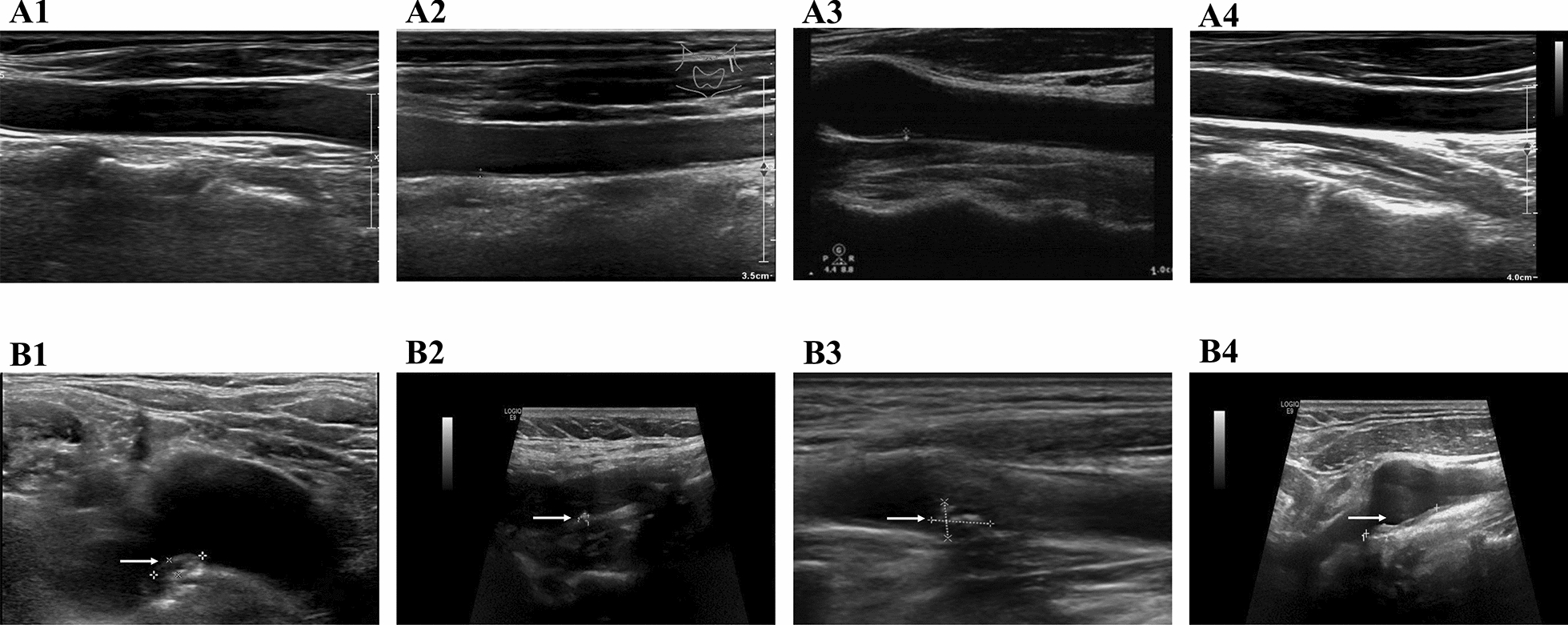
Illustration of carotid plaque growth patterns in different TyG quartile groups. 1,2,3 and 4 represent the four participants in each TyG quartile group. A1-A4 are carotid ultrasonography images at the baseline. B1-B4 correspond to the carotid ultrasonography images showing carotid plaque occurrence during follow-up. The white arrows in B1–B4 indicate the presence of the newly developed carotid plaque

A high-resolution B-mode ultrasound instrument examined three carotid artery areas with carotid ultrasonography on the subjects (internal carotid artery, bifurcating carotid artery, and common carotid artery). The Mannheim Carotid Intima-Media Thickness (cIMT) Consensus and the American Society of Echocardiography classified carotid plaque as cIMT lumen protrusion ≥1.5 mm [26]. A new carotid plaque was defined as the carotid plaque newly detected during the follow-up, whether it is stable or unstable. During the acquisition of the images, a strict quality control procedure was followed during carotid plaque measurement. Figure 2 illustrates carotid plaque growth patterns of different TyG quartile groups in the carotid ultrasonography images.

### Statistical analysis

Descriptive statistics were used to summarize information on general demographic characteristics. The statistical application SPSS was used for all analyses (version 21.0, SPSS software, Chicago, IL, USA). Continuous variables were presented using the median and interquartile range [M(P25, P75)], while categorical variables were expressed using frequency (constituent ratio).

Continuous variables were tested for normality before being compared with the *t*-test, Wilcoxon test, Friedman test, or one-way ANOVA; Categorical variables between groups were described as percent and were compared using the Chi-square test. All P-values were taken bilaterally for statistical inference, and *P* < 0.05 was considered a statistically significant difference.

Subjects were categorized according to the TyG index, and the baseline characteristics of subjects in each group were compared. Cox proportional hazards regression models were used to investigate the relationship between TyG and incident carotid plaque. The shortest follow-up time was 184 days, and the longest follow-up time was 1006 days. Subgroup analyses were also utilized to examine the robustness of the TyG index's relationship to carotid plaque.

## Results

### Baseline characteristics

Table 1 shows the baseline sociodemographic characteristics and medical parameters according to the quartile interval of the TyG index. A combination of 2370 research participants was evaluated at baseline, including 1381 males and 989 females. Participants with an elevated TyG index were more prone to be older males, former and current smokers, have diabetes, hypertension and dyslipidemia, or have higher BMI, SBP, DBP, FBG, TC, TG, and LDL-C. In contrast, HDL-C level was significantly lower than those with a lower TyG index (*P* < 0.001). Participants excluded from the analysis were slightly younger and had lower TC, TG, LDL-C, FBG, and TyG index (Additional file 1: Table S1).

**Table 1:** Baseline characteristics of the participants

Variables	TyG quartile				
	Quartile 1 (n = 593)	Quartile 2 (n = 592)	Quartile 3 (n = 593)	Quartile 4 (n = 592)	*p *value
TyG index	8.08 (7.93–8.21)	8.50 (8.41–8.60)	8.87 (8.78–8.96)	9.39 (9.21–9.71)	** < 0.001**
Gender (male/%)	223/37.6	326/55.1	391/65.9	441/74.5	** < 0.001**
Age (years)	46.00 (40.00–52.00)	48.00 (42.00–55.00)	49.00 (42.50–55.00)	50.00 (44.00–56.00)	** < 0.001**
BMI (kg/m^2^)	22.03 (20.29–24.26)	23.73 (21.86–25.60)	24.73 (22.77–26.73)	25.61 (24.01–27.45)	** < 0.001**
Dyslipidemia (n/%)	8/1.3	17/2.9	28/4.7	60/10.1	** < 0.001**
Diabetes (n/%)	2/0.3	15/2.5	29/4.9	98/16.6	** < 0.001**
Hypertension (n/%)	75/12.6	146/24.7	180/30.4	247/41.7	** < 0.001**
Smoking History					** < 0.001**
Never(n/%)	526/88.7	482/81.4	452/76.2	410/69.3	
Former (n/%)	15/2.5	21/3.5	33/5.6	33/5.6	
SBP (mmHg)	116 (106–127)	120 (110.25–132)	124 (114.5–135)	128 (119–138)	** < 0.001**
DBP (mmHg)	70 (64–78)	75 (67–83)	77 (70–86)	82 (75–89)	** < 0.001**
TC (mmol/L)	5.14 (4.49–5.78)	5.30 (4.74–6.03)	5.53 (4.95–6.18)	5.79 (5.09–6.65)	** < 0.001**
TG (mmol/L)	0.79 (0.68–0.89)	1.17 (1.06–1.28)	1.65 (1.49–1.82)	2.64 (2.23–3.36)	** < 0.001**
LDL-C (mmol/L)	3.12 (2.69–3.57)	3.35 (2.94–3.85)	3.55 (3.08–4.00)	3.71 (3.23–4.27)	** < 0.001**
HDL-C (mmol/L)	1.52 (1.32–1.74)	1.38 (1.17–1.58)	1.27 (1.10–1.44)	1.14 (1.01–1.29)	** < 0.001**
FBG (mmol/L)	5.07 (4.81–5.36)	5.29 (5.00–5.60)	5.40 (5.04–5.79)	5.73 (5.30–6.34)	** < 0.001**

Bold *P* values indicate significance

*TyG index* triglyceride-glucose index, *BMI* body mass index, *SBP* systolic blood pressure, *DBP* diastolic blood pressure, *TC* Total cholesterol, *TG* Triglyceride, *LDL-C* low-density lipoprotein cholesterol, *HDL-C* high-density lipoprotein cholesterol, *FBG* fasting blood glucose

### Relationship between TyG index and the probability of developing carotid plaques

**Fig. 3:**
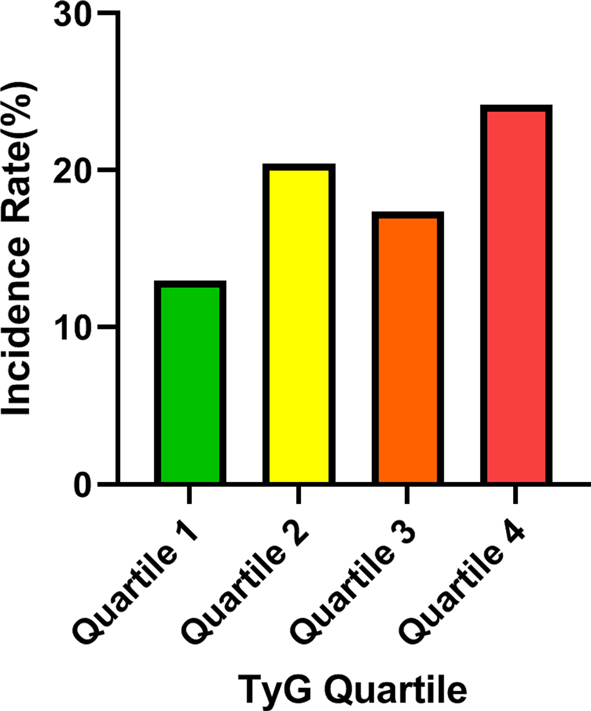
The incidence rate of carotid plaque during a 3-year follow-up

A total of 444 subjects (277 men and 167 women) developed carotid plaque during the 3 years follow-up, with a cumulative incidence rate of 20.1% in men and 16.9% in women (Table 2). The incidence of carotid plaque over the past three years was 18.7%, ranging from 17.3% in quartile 1 to 27.3% in quartile 2, 23.2% in quartile 3 to 32.2% in quartile 4, suggesting individuals with elevated TyG levels were more susceptible to suffer from carotid plaque (Fig. 3). In addition, older male subjects were more vulnerable to acquiring carotid plaque, and stratified analyses disclosed that subjects had a significantly higher risk of developing carotid plaque if they had a previous disease history (Table 2).

**Table 2:** Incidence rate stratified by sex, age, and chronic conditions

Categories	N/Total	Incidence rate (%)	Average follow-up time (days)	*p* value
Gender				0.051
Male	277/1381	20.06	473.79	
Female	167/989	16.89	477.37	
History of dyslipidemia				**0.007**
Yes	32/113	28.32	475.63	
No	412/2257	18.25	475.10	
History of diabetes				** < 0.001**
Yes	50/144	34.72	443.84	
No	394/2226	17.70	479.11	
History of hypertension				** < 0.001**
Yes	156/648	24.07	468.33	
No	288/1722	16.72	478.82	
Age (years)				** < 0.001**
18-	77/905	8.51	474.07	
45-	314/1326	23.68	481.09	
65-	51/134	38.06	442.37	
80-	2/5	40.00	418.00	
Total	444/2370	18.73		

Bold *P* values indicate significance

### The TyG index and the tendency to develop carotid plaque

The hazard ratio of carotid plaque occurrence in every TyG group was examined using univariate and multivariate Cox regression models (Table 3). Subjects who developed carotid plaque were predominantly male. The baseline age, BMI, TyG, SBP, DBP, TC, TG, LDL-C and FPG were substantially higher among those developing carotid plaque, while the baseline HDL-C level was lower. Table 3 shows the multiple analyses of TyG groups by adjusting for potential covariates. Compared with the lowest TyG group, subjects with TyG at quartile 2, 3, and 4 groups had HR (95% CI) of 1.590 (1.194–2.116), 1.423 (1.059–1.912) and 2.026 (1.535–2.675), respectively (*P* < 0.001). The correlation remained significant even after adjusting for age and gender (Model 1); for age, gender, and BMI (Model 2); and for age, gender, and other related variables (Model 3). Similar findings were shown in Table 4, where the TyG index was used as a continuous variable. We also observed a significant interaction of TyG index with gender and medical history of diseases in the risk of developing carotid plaque (Table 5).

**Table 3:** Risk factors of development of carotid plaque during 3-year follow-up

Variables	Univariate models		Multivariate models	
	HR (95%CI)	*P* value	HR (95%CI)	*P* value
Gender (male)	1.327 (1.095–1.608)	**0.004**	1.327 (1.068–1.650)	**0.011**
Age (years)	1.052 (1.042–1.062)	**< 0.001**	1.049 (1.039–1.060)	** < 0.001**
SBP (mmHg)	1.014 (1.009–1.020)	**< 0.001**	1.004 (0.997–1.012)	0.246
LDL-C (mmol/L)	1.072 (0.946–1.215)	0.275	1.054 (0.917–1.212)	0.456
HDL-C (mmol/L)	0.681 (0.496–0.933)	**0.017**	0.880 (0.585–1.324)	0.539
History of hyperlipidemia	1.376 (0.959–1.975)	0.083	1.073 (0.737–1.561)	0.713
History of hypertension	1.490 (1.226–1.811)	**< 0.001**	1.057 (0.811–1.377)	0.682
History of diabetes	2.207 (1.643–2.966)	**< 0.001**	0.717 (0.519–0.991)	**0.044**
BMI Classification				
Underweight (< 18.5)	Ref.	0.558	Ref.	0.144
Normal (18.6–24.9)	1.551 (0.690–3.484)	0.288	1.207 (0.532–2.741)	0.653
Overweight (25–29.9)	1.651 (0.730–3.734)	0.228	0.932 (0.401–2.166)	0.869
Obesity (> = 30)	1.831 (0.735–4.56)	0.194	1.003 (0.388–2.595)	0.995
TyG				
Quartile 1	Ref.	**< 0.001**	Ref.	**0.044**
Quartile 2	1.590 (1.194–2.116)	**0.001**	1.376 (1.021–1.854)	**0.036**
Quartile 3	1.423 (1.059–1.912)	**0.019**	1.083 (0.779–1.507)	0.634
Quartile 4	2.026 (1.535–2.675)	**< 0.001**	1.437 (1.007–2.052)	**0.046**

Bold *P* values indicate significance

*SBP* systolic blood pressure, *LDL-C* low-density lipoprotein cholesterol, *HDL-C* high-density lipoprotein cholesterol, *BMI* body mass index

**Table 4:** Risk of carotid plaque development according to baseline TyG categories

Models	Quartile 1 (n = 593)	Quartile 2 (n = 592)	Quartile 3 (n = 593)	Quartile 4 (n = 592)	Continuous TyG	*p for trend*
Crude HR(95% CI)	1.00	1.590 (1.194–2.116)	1.423 (1.059–1.912)	2.026 (1.535–2.675)	1.422 (1.224–1.653)	** < 0.001**
Model 1 HR(95% CI)	1.00	1.394 (1.045–1.860)	1.120 (0.829–1.514)	1.539 (1.155–2.051)	1.249 (1.061–1.469)	**0.008**
Model 2 HR(95% CI)	1.00	1.421 (1.064–1.897)	1.156 (0.854–1.567)	1.646 (1.224–2.214)	1.295 (1.095–1.531)	**0.003**
Model 3 HR(95% CI)	1.00	1.376 (1.021–1.854)	1.083 (0.779–1.507)	1.437 (1.007–2.052)	1.182 (0.955–1.462)	**0.044**

Bold *P* values indicate significance. Mode 1 was adjusted for age and gender. Mode 2 was further adjusted for BMI based on Model 1. Mode 3 was adjusted for SBP, LDL-C, HDL-C, history of hyperlipidemia, hypertension and diabetes based on Model 2

*SBP* systolic blood pressure, *LDL-C* low-density lipoprotein cholesterol, *HDL-C* high-density lipoprotein cholesterol

**Table 5:** Subgroup analysis of TyG index as a continuous variable

Variables	N	Unadjusted HR(95% CI)	Mode 1 HR(95% CI)	Mode 2 HR(95% CI)	*P* for interaction
Gender					**0.001**
Female	989	1.951 (1.510–2.519)	1.588 (1.202–2.098)	1.544 (1.080–2.208)	
Male	1381	1.117 (0.913–1.366)	1.099 (0.896–1.347)	1.149 (0.893–1.478)	
Age (years)					0.545
< 45	905	1.503 (1.079–2.092)	1.276 (0.882–1.845)	1.247 (0.775–2.005)	
≥ 45	1465	1.335 (1.121–1.591)	1.229 (1.024–1.475)	1.207 (0.964–1.511)	
Disease history					**0.034**
No	1619	1.530 (1.225–1.911)	1.368 (1.078–1.735)	1.381 (1.038–1.837)	
Yes	751	1.094 (0.868–1.379)	1.109 (0.872–1.411)	1.229 (0.944–1.599)	

Bold *P* values indicate significance. Mode 1 was adjusted for age and gender. Mode 2 was further adjusted for BMI, SBP, LDL-C, HDL-C, history of hyperlipidemia, hypertension and diabetes based on Model 1

Disease history included hypertension, dyslipidemia and diabetes. The presence of any of such diseases is considered to be having a history

*BMI* body mass index, *SBP* systolic blood pressure, *LDL-C* low-density lipoprotein cholesterol, *HDL-C* high-density lipoprotein cholesterol

### Sensitivity analysis

We utilized a sensitivity analysis to assess the stability of the relations. The association between the TyG index and the risk of carotid plaque was not materially altered after excluding participants who developed carotid plaque within the first 9 months of the follow-up, participants with hypertension, diabetes, or dyslipidemia at baseline (Additional file 1: Table S2).

## Discussion

This population-based study demonstrated a strong correlation between participants' elevated baseline TyG index and their probability of acquiring carotid plaque in a Chinese population. The association remained significant even after adjusting for confounders, showing that the TyG index represents an independent risk factor for the development of carotid plaque.

### TyG index as a suitable indicator for assessing cardiovascular risk factors

The TyG index is a viable alternative to measure IR. It correlates with elevated cholesterol and glucose levels, thus being a suitable metric for assessing the relationship between IR and cardiovascular risk factors [27]. It has been shown that there was a significant linear correlation between the TyG index and cardiovascular risk biomarkers, which were commonly used for predicting atherosclerosis, dyslipidemia, renal vascular damage, and dysglycemia [28]. According to a large-scale study conducted in China, the TyG index was easier to use and more suited for identifying metabolically unhealthy individuals and those with a high probability of developing cardiometabolic illness [29]. The TyG index has been linked in research to various cardiometabolic disorders. A 9-year prospective investigation has shown that the TyG index independently predicted the new cases of hypertension [19]. Another prospective cohort study has indicated that an elevated TyG index precedes and significantly predicts future ischemic heart disease [30]. Additionally, the TyG index was an important marker for identifying type 2 diabetes and obesity [31, 32]. As an alternative biomarker of IR, the TyG index could shed new light on pathophysiological changes, and further studies are needed to elucidate the underlying biological mechanisms.

Carotid plaque incidence was increasing around the globe, with younger people suffering from it [9]. Significant financial and health damage has resulted from carotid plaques due to the need for medication and surgical treatment. Therefore, it was essential to determine the risk factors associated with carotid plaques to prevent and intervene. A previous cross-sectional study described a significant correlation between the TyG index and carotid plaque occurrence among diabetic patients aged 40–70 years [15]. A prospective cohort study of 6955 participants with 4264 (61.3%) males reported that a higher prevalence of carotid arteriosclerosis in public was linked to an elevated TyG index [9]. However, another study indicated that a high TyG index was significantly related to an increased risk of arterial stiffness and nephric microvascular damage, though not artery hypertrophy or carotid plaque [16]. Therefore, this study used a cohort supplemented with the TyG index at baseline examination to evaluate the correlation between the TyG index and carotid plaque formation.

### Possible mechanisms underlying the association between TyG index and carotid plaque

Even though the underlying cause of the association between TyG and the prevalence of carotid plaque was unknown, this could have implications for IR. Numerous potential mechanisms may underlie the association between IR and metabolic disorders such as ASCVDs. IR mediates systemic inflammation, oxidative stress, and vascular remodeling by promoting endothelial dysfunction and leading to the release of reactive oxygen species [15]. IR impairs the nitric oxide (NO) synthesis system in the vascular endothelium and causes the endothelial cells to lose their normal physiological state and become dysfunctional [33]. As a result, IR plays a crucial part in fostering the growth of carotid plaque. The Nitric oxide (NO) production system in the vascular endothelium is compromised by IR, which also causes the endothelial cells to lose their normal physiological state and become dysfunctional [34]. As a result, IR plays a crucial part in encouraging the development of carotid plaque and atherosclerosis. What’s more, IR can induce an IFNγ-macrophage pathway, which may potentially contribute to carotid plaque progression [34]. IR was significantly linked to the onset of coronary atherosclerosis [35]. Another study showed a high incidence of carotid plaque in insulin-resistant patients [36]. Therefore, IR was an independent risk factor for cardiometabolic diseases [37, 38]. Numerous investigations have revealed a link between the TyG index and IR-related metabolic disorders. According to cross-sectional research, the TyG index was a substitute for IR [24]. A study in Mexico verified that the TyG index has a high concordance with HOMA-IR in young adults [39]. The TyG index helps screen for IR status as a diagnostic test for IR.

### Public health implications

It was known that high TG, FBG, and insulin resistance are all underlying causes of coronary events, and they were closely associated with preclinical cardiovascular organ damage, coronary artery lesions, and poor prognosis. In many clinical circumstances, the TyG index was a well-accepted indicator of the onset of coronary atherosclerosis [40]. Research revealed a correlation between an increased TyG index and the frequency and severity of artery stenoses, indicating people at high risk of artery stenoses [41]. In asymptomatic populations, elevated incidence of coronary calcium progression was statistically significantly associated with an elevated TyG index [42]. Two Korean studies showed that the TyG index is related to coronary calcium and arterial stiffness [43, 44]. In one of these studies, Lee et al. demonstrated a statistically significant association between TyG index and arterial stiffness as determined by the brachial-ankle pulse wave velocity (baPWV). They discovered that compared to HOMA-IR, the TyG index was more significantly related to elevated arterial stiffness [44]. Similarly, the results of another research indicated that the TyG index was independently and positively associated with baPWV among a Chinese hypertensive cohort, especially in men [45]. All these studies point to the possibility of using the TyG index as a biomarker to evaluate arterial vascular lesions.

Our findings suggested that the TyG index positively predicts carotid plaque development. The TyG index may be employed as a tool for early diagnosis of carotid plaques and even atherosclerosis conditions. Monitoring the community's TyG index can identify individuals with IR before the onset of significant disease, thus providing physicians with valuable clinical information and an important basis for prevention strategies. Using the TyG index in routine physical examinations can effectively reduce the risk of carotid plaque and the severity of coronary artery disorders. Our study demonstrates the need for prevention strategies targeting key populations with relatively high TyG index. In addition, for the entire population, measures such as lifestyle improvement can be used to reduce TyG.

## Strengths and limitations

The main advantage of our study was the 3-year longitudinal cohort study. This prospective study extended the investigation to determine the time sequence between the TyG index and the probability of carotid plaque. Moreover, the follow-up procedures for incident carotid plaque were accurate, the information was obtained with advanced diagnostic techniques, and the population-based sample size was relatively large. Selection bias was investigated by comparing the baseline characteristics of included and excluded participants.

However, several limitations in the current study also need to be considered. Firstly, we found some differences between those included and not included, so the selective bias could not be avoided. Follow-up work such as inverse probability weighting (IPW) still needs to be conducted to reduce the impact of particular bias. Second, we only assessed the presence or absence of plaque components, whereas assessing carotid plaque stability may provide more detailed information. Nonetheless, a relatively simple assessment of the existence of carotid plaque has provided critical information on the risk of cardiovascular events. Third, the populations included in this study were employed with the requirement of routine annual physical examinations, the extent to which this finding applies to those who were unemployed needs to be further explored. Fourth, our study was also limited to using self-reported conditions. Objective health status measures would have been preferable. However, studies comparing self-reported chronic diseases with medical records have shown acceptable levels of agreement [46]. Fifth, our study lacks information about lipid-lowering medications, such as statins, ezetimibe, PCSK9 inhibitors, and inclisiran, which may influence the incidence of carotid plaque. However, we excluded people with stroke, coronary artery disease, or carotid plaque at baseline, who were most likely to use lipid-lowering medications, thus reducing the impact of the lipid-lowering medications on the study. A final limitation is the relatively short follow-up time. Further research is required to understand the mentioned issues.

## Conclusions

To summarize, our research revealed that a higher TyG index was associated with an elevated incidence of carotid plaque in the Chinese population. These findings suggested that physicians may monitor the TyG index closely during routine check-ups since it may aid in the early recognition and treatment of carotid plaques, atheromatous, and cardiovascular disorders. There may be merit in further studies to find out if lowering the TyG index might prevent the progression of carotid plaques.

## Supplementary Information

**Additional file 1: Table S1**. Baseline characteristics of the included and excluded populations. **Table S2**. Sensitivity analysis on the association between TyG index and carotid plaque.

## Data Availability

The datasets used and/or analyzed during the current study available from the corresponding author on reasonable request.

## Abbreviations

ASCVD: Atherosclerotic cardiovascular disease
CAD: Coronary artery disease
IMT: Intima-media thickness
IR: Insulin resistance
T2DM: Type 2 diabetes mellitus
HOMA-IR: Homeostasis model assessment of insulin resistance
TyG: Triglyceride-glucose
TG: Triglyceride
FBG: Fasting blood glucose
CHD: Coronary heart disease
BMI: Body mass index
SBP: Systolic blood pressure
DBP: Diastolic blood pressure
TC: Total cholesterol
LDL-C: Low-density lipoprotein cholesterol
HDL-C: High-density lipoprotein cholesterol
HR: Hazard ratio
CI: Confidence interval
baPWV: Brachial-ankle pulse wave velocity
IPW: Inverse probability weighting
