# Supplementary material for: Association between the triglyceride-glucose index and carotid plaque incidence: a longitudinal study
Source: Cardiovasc Diabetol. 2022 Nov 15;21:244. doi: 10.1186/s12933-022-01683-6 (PMC9667568; doi:10.1186/s12933-022-01683-6)
Supplement: Supplementary file 1 — Additional file 1: Table S1. Baseline characteristics of the included and excluded populations. Table S2. Sensitivity analysis on the association between TyG index and carotid plaque. [file 12933_2022_1683_MOESM1_ESM.docx]

**Table S1.** Baseline characteristics of the included and excluded populations

| **Variables** | **Participants included** | **Participants excluded** | ***p* value** |
| --- | --- | --- | --- |
| TyG index | 8.73±0.59 | 8.64±0.59 | **< 0.001** |
| Gender (male/%) | 54.8 | 58.3 | **0.016** |
| Age (years) | 48.68±10.30 | 47.30±10.82 | **< 0.001** |
| BMI (kg/m2) | 24.24±3.14 | 24.02±3.29 | **0.023** |
| SBP (mmHg) | 123.70±16.63 | 124.25±17.12 | 0.267 |
| DBP (mmHg) | 76.85±17.20 | 76.58±11.36 | 0.535 |
| TC (mmol/L) | 5.51±1.05 | 5.19±0.99 | **< 0.001** |
| TG (mmol/L) | 1.67±1.13 | 1.57±1.59 | **0.005** |
| LDL-C (mmol/L) | 3.47±0.75 | 3.13±0.72 | **< 0.001** |
| HDL-C (mmol/L) | 1.35±0.31 | 1.36±0.33 | 0.149 |
| FBG (mmol/L) | 5.51±0.99 | 5.33±1.04 | **< 0.001** |
| Dyslipidemia (n/%) | 2.0 | 4.8 | **< 0.001** |
| Diabetes (n/%) | 2.8 | 6.1 | **< 0.001** |
| Hypertension (n/%) | 13.9 | 27.3 | **< 0.001** |

**Note:** Bold *P* values indicate significance.

**Abbreviations**: TyG index, triglyceride-glucose index; BMI, body mass index; SBP, systolic blood pressure; DBP, diastolic blood pressure; TC, Total cholesterol; TG, Triglyceride; LDL-C, low-density lipoprotein cholesterol; HDL-C, high-density lipoprotein cholesterol; FBG, fasting blood glucose.

**Table S2**. Sensitivity analysis on the association between TyG index and carotid plaque

| Sensitivity analysis | HR (95% CI) | | | | ***P* value** |
| --- | --- | --- | --- | --- | --- |
|  | Quartile 1 | Quartile 2 | Quartile 3 | Quartile 4 |  |
| Analysis 1 | Ref. | 1.501 (1.044-2.157) | 1.111 (0.754-1.638) | 1.726 (1.195-2.492) | **0.006** |
| Analysis 2 | Ref. | 1.417 (1.059-1.895) | 1.155 (0.851-1.567) | 1.645 (1.221-2.215) | **0.003** |
| Analysis 3 | Ref. | 1.336 (0.993-1.796) | 0.991 (0.720-1.363) | 1.443 (1.063-1.958) | **0.014** |
| Analysis 4 | Ref. | 1.348 (1.002-1.814) | 1.131 (0.828-1.546) | 1.571 (1.161-2.127) | **0.012** |

**Note:** Bold *P* values indicate significance; Multivariable model adjusted for age, sex, and BMI. Analysis 1 excluded participants with events occurring within the first 9 months of follow-up; Analysis 2 excluded participants with hypertension at baseline; Analysis 3 excluded participants with diabetes at baseline; Analysis 4 excluded participants with dyslipidemia at baseline.

**Abbreviations**: BMI, body mass index.
